# Application of CO_2_ Gas Hydrates as Leavening Agents in Black-and-White Cookies

**DOI:** 10.3390/foods12142797

**Published:** 2023-07-23

**Authors:** Ann Mary Kollemparembil, Shubhangi Srivastava, Viktoria Zettel, Bernhard Gatternig, Antonio Delgado, Mario Jekle, Bernd Hitzmann

**Affiliations:** 1 Department of Process Analytics and Cereal Science, University of Hohenheim, 70599 Stuttgart, Germany; 2 Institute of Fluid Mechanics (LSTME), FAU Erlangen-Nüremberg, 91058 Erlangen, Germany; 3 Process Engineering and Circular Economy, University of Applied Sciences Weihenstephan-Triesdorf, 85354 Triesdorf, Germany; 4 German Engineering Research and Development Center LSTME Busan, Busan 46742, Republic of Korea; 5 Department of Plant-Based Foods, Institute of Food Science and Biotechnology, University of Hohenheim, 70599 Stuttgart, Germany

**Keywords:** acrylamide, ammonium bicarbonate, baking, black-and-white cookie, gas hydrate, leavening agent

## Abstract

In this unprecedented study, the application of CO_2_ gas hydrates (GH) as a leavening agent to produce black-and-white cookies by replacing ammonium bicarbonate is investigated. Ammonium bicarbonate, the principal leavening ingredient in black-and-white cookies, has been linked to the creation of a carcinogenic substance known as acrylamide. Three distinct GH concentrations, 20%, 40%, and 50%, were utilized to determine the necessary amount to obtain a good leavening effect. However, the abrupt reduction in temperature brought on by the addition of GH had an inadmissible effect on the cookie dough. Consequently, an innovative kneading method carried out in a closed mixing unit at a high temperature was developed. The specific volume of the cookies when employing 50% GH as a baking agent was more than half that produced when using ammonium bicarbonate. In the cookies with GH, the springiness and hardness, which are the quality-determining textural characteristics of the pastry, remained within an acceptable range. The amount of acrylamide was reduced from 24.8 µg/Kg to around 18 µg/Kg by this research. Therefore, the presented study demonstrates the possibility of using CO_2_ GH as a leavening agent in black-and-white cookies and in other products for a healthier future.

## 1. Introduction

The term “baked products” refers to foods produced using recipes that primarily use or contain significant amounts of wheat or other cereal flour. These foods are combined with other ingredients, which are formed into distinct shapes, and then proceed through a heat-processing step that involves removing the moisture in a baking oven. A variety of food items, including bread, cakes, pastries, cookies, and crackers, are referred to as “baked products” [1].

Based on macroscopic observations, three major changes can be defined during baking. First, the formation of gases from the chemical leavening agents and water vaporization causes the product’s thickness to grow. Second, an open porous structure forms as the product weight falls because of drying. Finally, the browning of the cookies happens as a result of sugar caramelization and the non-enzymatic Maillard reaction, which involves the reaction between reducing sugars and proteins [2,3].

The structure, rheology, and other physical characteristics of baked goods, along with other ingredients like sugar and fat, are largely determined by the starch and protein content of the mixture. Because of the gelatinization and pasting of the starch and the coagulation of the egg protein during baking, the dough structure sets. In contrast to bread, where these components make up the fundamental structure, gluten proteins act as a binder in cookies. Fats help with the gas retention capacity of the dough, improving heat transfer while baking and providing the final products with a soft, moist mouthfeel [4]. Sucrose alters the hydration characteristics of proteins and restricts the amount of water that is available to starch granules, which results in starch gelatinization and protein denaturation [5]. Because it contains a lot of protein, eggs are a staple in the food processing industry [6]. They are made up of egg white and yolk, each of which has a different function in food systems [7]. In aerated systems, such as those in bakeries, egg whites are frequently employed as a foaming agent because of their outstanding reputation for producing a perfect structure and a large amount of product [8]. The capacity of eggs to coagulate and create gels while being heated is another advantageous quality for baking systems. Fats help in the integration of air into the dough, improving heat transfer throughout baking and contributing a moist mouthfeel and a soft consistency to the final products [9].

Due to the significant amount of flour, they contain, black-and-white cookies cannot be classified as a specific type of pastry. It can be differentiated from bread dough by the addition of eggs, fat, milk, and high amounts of sugar. Even though the ingredient mixture has the potential to be referred to as a dough, two steps of mixing, including the pre-batter creation, transform it into a soft and creamy substance with a lower gluten concentration. Consequently, a black-and-white cookie can be classified as a sweet shortcake.

Shortcakes and other bakery goods belong to a class of foods where the interactions between ingredients and thermal processing encourage the development of acrylamide [10,11]. The items created with the baking agent ammonium bicarbonate frequently have the greatest amount of acrylamide. The baking agents, reducing sugars, and asparagine, which come from cereals, milk, or eggs, are the elements in shortbread that have an impact on acrylamide formation [12]. The IARC classifies acrylamide, a known neurotoxic chemical, as “probably carcinogenic to humans” [13]. Its discovery in heat-processed foods generated alarm around the world. Consumers today like goods with clean labeling. Therefore, one of the primary goals of the food sector is to reduce compounds that have an adverse impact on health.

The baking agent, ammonium bicarbonate (E number 503), breaks down into ammonium and CO_2_ during baking, which helps cookies spread widely and create an interior structure [14,15,16]. Another role of ammonium bicarbonate is that it helps give black-and-white cookies their signature flavor.

Gas (guest) molecules are trapped in hydrogen-bonded water molecules to form gas hydrates (GH): non-stoichiometric solids that resemble ice. High pressure and low temperature are typical conditions for their development, with Van der Waals forces joining host and guest molecules [17,18]. The water molecules can arrange into symmetrical chambers (cages) with regular pentagonal and hexagonal faces [19]. Mechanical and chemical methods are routinely employed to promote GH growth. Over the past ten years, amino acids have taken on a greater role as additives in investigations of gas hydrates. They can perform electrostatic interactions with water. Most significantly, they are biodegradable and environmentally friendly [20].

The use of carbon dioxide was approved as E 290 in food bakery for production. There is no threshold to the acceptable daily intake, with no established side effects or dietary restrictions on this gas. Hence, the production of CO_2_ GH can be referred to as clean technology. The key benefits of GH include a lack of extra chemical components, the ability to control the process’s disintegration by freezing the dough, and its lack of contact with the end product’s sensory qualities. If used effectively, these CO_2_ GHs can take the place of current technologies that are used to produce various food items, including freeze-drying, reverse osmosis, and heat evaporation [21]. Leucine, methionine, and lecithin operate as the optimum sequence of promoters that aid in the creation of the most stable CO_2_ GH, according to Srivastava et al. [22]. However, the possibility of controlling and changing biscuit quality through the use of GH in cookie baking has not yet been investigated and is a novel concept in the food sector. The customer preference for clean-labeled meals is steadily growing, pushing the food industry to choose novel and environmentally friendly raw ingredients. Hence, this innovative study to reduce the acrylamide content for a healthier diet is of the utmost importance. In order to address the issues raised by the current leavening agent, this study looks into the use of GH as a novel leavening agent in black-and-white cookies and analyzes the quality of the cookies [23] by measurements of specific volume, moisture, hardness, springiness, and pore size analysis.

## 2. Materials and Methods

### 2.1. Production of GH

In order to produce GH, 500 mL of cold, deionized water containing promoters, 0.5 g of each leucine and methionine, and 5 g of lecithin [23] were added to the reactor. Carbon dioxide gas was introduced through the bottom valve of the reactor. To operate the system as a bubble column, the gas cylinder was opened until the optimal pressure within the reactor was reached (36–37 bars), and the upper vent valve on the reactor’s lid was kept open so that there would be the constant bubbling of gas in the water. Once the gas molecules were dissolved at a low temperature (0–1 °C), the synthesis of GH commenced with nucleation and lasted for three hours. The CO_2_ GH was produced in a reactor installed at the Department of Process Analytics and Cereal Science, University of Hohenheim, Stuttgart, Germany, in collaboration and assistance for installation with the Institute of Fluid Mechanics, FAU Erlangen-Nuremberg, Erlangen, Germany.

### 2.2. Production of Black-and-White Cookies

**Figure 1:**
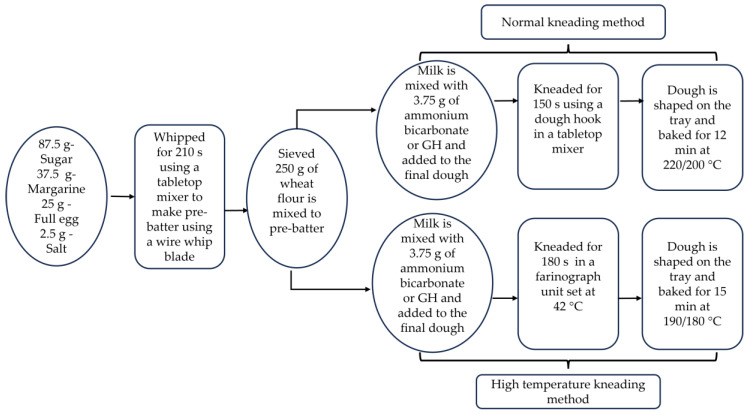
Schematic representation of the normal and high temperature kneading (HTK) method.

The recipe for black-and-white cookies was standardized by varying the ingredients and baking conditions. Different trials were conducted to obtain the most suitable recipe. The raw materials that were used to produce the cookies comprised 250 g of wheat flour type 550 (Rettenmeier Mühle GmbH, Horb am Neckar, Germany) and 37.5 g of margarine (Walter Rau Lebensmittelwerke GmbH, Hilter am Teutoburger Wald, Germany). A total of 112.5 mL of milk was prepared by mixing 21.5 g of skimmed milk powder (TSI Consumer Goods GmbH, Zeven, Germany) with 91 mL of lukewarm tap water. The required 25 g of egg (Hagenauer Hof KG, Neuhausen, Germany), 2.5 g of salt (Südwestdeutsche Salzwerke AG, Heilbronn, Germany) and 87.5 g of sugar (BÄKO-Zentrale Süddeutschland eG, Ladenburg, Germany) were obtained from a local market. To produce the standards, 3.75 g of ammonium bicarbonate (Gebrüder Neeb GmbH & Co. KG, Graefelfing, Germany) was used as a leavening agent. For the cookie production, all ingredients were kept at room temperature, at around 22 °C, before mixing. The dough production was made in two steps which included pre-batter production and a later mixing stage, as represented in Figure 1. Eggs, sugar, salt, and margarine were whipped for 210 s using a tabletop mixer (HOBART GmbH, Offenburg, Germany) and a wire whip blade to form the pre-batter. Sieved flour and the leavening agent dissolved in milk were added to the pre-batter, followed by kneading for 150 s using a dough hook in the same mixer. The dough was subsequently shaped and pressed into the desired form with a commercial pastry bag onto baking sheets and was baked for 15 min in a baking oven (Wachtel GmbH, Hilden, Germany) that was set at 190 °C for the top and 180 °C for the bottom heat. The baked cookies following the standard procedure are named ‘STD’ in the results and discussion.

The standard production methods were altered for the incorporation of GH by substituting ammonium bicarbonate. To account for the water in GH, which contains 85% water, the water in the milk powder mixture was reduced in proportion to the amount of GH added. The baker’s percentage was used to calculate the percentage of GH, which could be expressed as a percentage of the flour weight, with the flour weight being considered at 100%. The maximum amount of GH that could be added to the dough was calculated at 50% due to the water content of GH and the amount of water that could be removed from the recipe by substituting milk for milk powder. After adding flour to the pre-batter, GH was added to the dough and mixed in a standard tabletop mixer. Baking was conducted under the same conditions as the standard. Cookies baked with GH are named after the percentage of GH added to the cookie batter. Another set of cookies with no leavening agent was made as a control to compare the leavening effect of the rising agent.

To improve the baking results, the kneading method for incorporating GH into the cookie dough was modified. After the production of the pre-batter in a normal mixer, the mixing with flour was conducted in a farinograph (Brabender GmbH & Co. KG, Duisburg, Germany) mixing unit with temperature control. The temperature of the mixing unit was maintained at 42 °C. To prevent further gas escape into the environment, the mixing unit was sealed with duct tape. Once the pre-batter, flour, and milk were mixed for 10 s, GH was added to the unit for 180 s. The cookie dough was pressed onto the baking sheets. Baking was conducted for 12 min in the oven at 220 °C for the top heat and 200 °C for the bottom heat. The abbreviation of high-temperature kneading, HTK, is used to specify the cookie samples baked following this method. Batches of cookies were made without any leavening agent using the HTK method as a control for comparative study.

### 2.3. Analysis of Black-and-White Cookies

The measurements of different cookie characteristics were analyzed to compare the standard cookie made with ammonium bicarbonate and the cookie made with GH. All analyses were carried out in triplicate, and the data were reported as the means ± standard deviation. The methods followed for measuring the characteristics of cookies included moisture analysis, volume analysis, pore analysis, and texture profile analysis.

#### 2.3.1. Moisture Analysis

The standard cookies and the cookies made with GH were checked for their moisture content using an infrared moisture analyzer (Kern and Sohn GmbH, Balingen, Germany).

#### 2.3.2. Volume Analysis

Using a volume analyzer, the cookies’ volume was determined (Stable Microsystems, VolScan Profiler 600, Godalming, Surrey, UK). For product support, a flat disc-shaped geometry and a vertical skewer were employed to offer upright stability without harming the sample structure. The product was automatically weighed. In total, 400 data points made up each interval, giving the product a thorough profile. Equation (1) was used to obtain the specific volume:

Specific volume (mL/g) = Volume of cookie (mL)/Mass of cookie (g)(1)

#### 2.3.3. Texture Profile Analysis

The measurement of the hardness (N) and springiness (%) of the cookie was performed using a texture profile analyzer (TA-XT2, Stable Microsystems, Godalming, Surrey, UK) with an AOAC cylindrical acrylic probe (25.4 mm diameter, 35 mm tall). The crust of the cookie was removed with a knife before the measurement with a texture analyzer. During the experiment, the product was subjected to a compressive force by a probe two times according to the AACC [24] standard method to provide an understanding of the behavior of samples when chewed, called the ‘two bite test’.

#### 2.3.4. Pore Size Analysis

A pore scanner (Hp scan jet 5590, Düsseldorf, Germany) linked to inbuilt software (“Gebäck analyse version 1.4”, developed by University of Applied Sciences and Arts Ostwestfalen-Lippe, Lemgo, Germany) with Oracle Virtual Toolbox 6.1 was used to estimate the number and area of the pores in the cookie. The pore analysis software categorized the quantity of the pores into five groups based on their size, which ranged from extremely small to extremely large.

#### 2.3.5. Color Measurements

By using MATLAB^®^ software (R2022a), color measurements were made by computer vision-based image analysis. A digital image acquisition system consisting of a color digital camera with daylight fluorescent lamps was used to capture images of the cookies. Images were taken and saved in JPEG format on a computer. Using a MATLAB^®^ algorithm, the L*a*b* values were calculated from the image of the crust of the cookies. L* is the luminance or lightness component, which ranges from 0 to 100, and parameters a* (from green to red) and b* (from blue to yellow) are the two chromatic components, which range from −120 to 120. 

#### 2.3.6. Analysis of the Acrylamide Content

The acrylamide content was evaluated externally by the Core Facility of the University of Hohenheim and by their internal standard. These samples were dried prior to testing to eliminate the moisture content by placing them in a drying oven (Memmert GmbH + Co. KG, Schwabach, Germany) at 60 °C for 20 h. The samples were homogenized and were analyzed fresh or stored at −20 °C until analysis. An internal standard was added (1 µg D3-Acrylamid), and the samples were extracted by means of a homogenizer. The LC MS/MS (AB Sciex QTRAP 5500 LC-/MS/MS, Sciex, Concord, ON, Canada) was used, utilizing a well-managed Agilent 1290 G4229 Binary Pump (Agilent Technologies, Inc., Santa Clara, CA, USA). The sample extraction was performed by defatting 1 g of the sample with 50 mL of n-Hexane. An internal standard of 1 µg D3-Acrylamide was added, followed by its extraction with 20 mL of water. Protein depletion was conducted by methanol. Solid phase extraction was performed for purification using a CHROMABOND ABC18 column. Finally, the tenfold concentration was conducted under the flow of nitrogen gas. Chromatographic separation was achieved on the Phenomenex Luna^®^ Omega 1.6 um polar C18 (LC 150 × 2.1 mm) column (Phenomenex, Torrance, CA, USA). The detection of analytes was conducted using a multiple-reaction mode (MRM). The dwell time for each MRM transition was 0.20 s.

### 2.4. Statistical Analysis

All different cookie doughs were prepared in triplicate. The number of measurements per dough for each experiment was conducted, as mentioned in each method section. Furthermore, each type of cookie was baked in triplicate with 6 cookies per batch, and the experiments were performed as mentioned in each method section. A variance analysis (one-way ANOVA, *p* ≤ 0.05, Tukey test) was performed using Excel (2019).

## 3. Results and Discussion

### 3.1. Physical Characteristics of Black-and-White Cookies

In Table 1, the results of the specific volume, moisture content, hardness, and springiness of the cookies are presented. The specific volume of each cookie baked under varied conditions was calculated using Equation (1). As one can see, the conventional cookies baked with ammonium bicarbonate had a maximum specific volume, as can be seen in Table 1. Standard black-and-white cookies were baked at normal and high temperatures with specific volumes of 2.85 ± 0.04 mL/g and 2.75 ± 0.06 mL/g, respectively. When compared to the volume of the cookie without a leavening agent, Table 1 showed that the addition of 20%, 40%, and 50% GH had no significant influence on the volume of the cookie. Each of these samples had a specific volume that fell between 0.7 and 0.9 mL/g. However, the volume of cookies baked with 50% GH increased by kneading at 42 °C. The volume of GH-containing cookies made under improved conditions was 1.8 ± 0.05 mL/g, which was more than twice the cookies containing the same amount of GH baked under normal conditions.

The initial mixing step in the formation of black-and-white cookies was the mechanical whipping of the pre-batter in a tabletop mixer. Many diverse methods, including mechanical whipping, are used to create foam. In a planetary mixer, an impeller generates an axial flow pattern that forces any present gas or air to swell on the surface of the frothy liquid. As air is drawn into the foamy liquid, bubbles may break or group together. Tabletop planetary mixers can create highly stable foams that have very small bubble diameters between 0.01 and 1 mm [25,26]. According to Jha et al. [27], doughs’ ability to dissolve CO_2_ rises both under pressure and when the headspace contains CO_2_. The gas produced from GH becomes soluble in the dough without escaping into the environment when the second step of mixing the dough is carried out inside the farinograph mixing unit at a high temperature and with less interaction with the outside environment.

The measured air volume percentages considerably increased over the course of two min as a result of aeration, which raises the viscosity of the batter and uses energy to produce a new bubble surface area. Six min of aeration are used as part of standard industrial mixing before baking [28]. Hence, the amount of time required for the aeration of the dough during the pre-batter manufacturing phase and the second mixing phase together accounts for the duration of this study. Since hydrogen bonds are weaker than disulfide bonds, it has been hypothesized that low temperatures increase and strengthen them. However, the greater mixing temperature for disulfide bonds aids in stabilizing and fortifying the network structure [29].

The gluten network formation must be kept to a minimum in semisweet confectionery dough in order to keep the dough extensible enough to be easily sheeted but not too elastic to prevent the biscuit from shrinking after molding and baking [30]. An increased dough temperature has an impact on density as well. This is because nearly no fat crystals are present when the dough temperature is over 35 °C. Triglyceride crystals in cake batter and bread dough are able to stabilize air bubble interfaces [31]. In this regard, speeding up the mixing process could result in better fat crystal spatial distribution and lead to the better stabilization of air bubbles with a reduced dough density [30]. On the other hand, air bubbles could burst as a result of fat melting at temperatures above 35 °C, increasing the dough’s density. Keeping the mixer unit at 42 °C results in a dough temperature of 32 °C, which aids in the melting and homogeneous dispersion of fat molecules inside the dough. The addition of GH lowers the dough temperature to below 15 °C.

During baking, a few significant physicochemical changes that alter the dough’s components take place. Beginning at a temperature above 85 °C first causes a decline in protein solubility. Second, drying the cookies restricts starch denaturing, which essentially takes place in the first part of the baking time. Third, in the last two-thirds of baking time, a decline in the content of reducing sugar, which is related to the surface color, is observed. The melting of fat, which depends on fat polymorphism and the amount of solid fat, occurs between 15 and 40 °C and further complicates the process [32]. From the experiments conducted by Srivastava et al. [23] with the addition of GH, promoters lead to half the specific volume when compared to standard wheat bread. From this, it can be understood that modified kneading conditions like increased temperature could enhance the results without any further incorporation of additives.

It is clear from Table 1 that the lowest moisture contents of 30.12 ± 0.3% and 29.58 ± 0.4% were found in the conventional black-and-white cookies with normal kneading and the improvised black-and-white cookies with improvised kneading, respectively. The moisture content of the cookies containing 20% and 40% GH shows that the amount of GH in the dough is directly correlated with the amount of moisture in the baked cookie. The cookies baked with 50% GH, mixed with a normal mixer, and baked under normal conditions had the highest moisture content of 31.10 ± 0.62%. At both kneading conditions, the moisture content of cookies made without a leavening agent was higher than that of regular cookies but lower than that of cookies made with GH. Hence, it can be concluded that the increased water content caused by the addition of GH caused the moisture percentage to become elevated.

The final product’s quality is significantly influenced by the cookie’s hardness. The initial compression of the product’s peak force was used to calculate the product’s hardness [33]. The cookie with the least hardness was the one made with standard recipe cookies, as shown in Table 1. A standard cookie baked under normal conditions has a hardness of 5 ± 1.08 N. However, a standard cookie baked with improved kneading has a hardness of 8.41 ± 0.88 N. This rise can be brought on by the higher baking temperature used in the improvised method. Under any baking conditions, cookies without a leavening agent can have a very high degree of hardness. Although the hardness value drops as the percentage of GH addition rises, these values are still above the acceptable limit. Cookies with a GH content of 20% had the hardest surface, measuring 45.52 ± 4.26 N, whereas cookies with a GH content of 50% had the softest surface, measuring 38.76 ± 1.89 N. However, the cookies that contained 50% GH and which were prepared under the new conditions had a final hardness of 24.25 ± 2.18 N. When compared to the standard cookie, this value of hardness in the cookie is extensively high. The higher baking temperature utilized in the improvised cookie-making technique could be presumed to be the cause of this. Furthermore, the lowest value of hardness of wheat bread after the addition of GH was found to be threefold that of standard bread [23]. Therefore, the addition of GH had adverse effects on the hardness profile of the baked food materials. In order to make the cookie softer and consequently reduce its hardness, CO_2_ from GH that is dissolved in the dough has to be released during baking. When more gas is dissolved in the dough, a higher amount of gas is released during baking. Since the second stage of mixing is conducted at a higher temperature and in a closed environment, more CO_2_ can be dissolved in the dough. Therefore, a stronger impact of CO_2_ in the headspace on the amount of spreading and also on comparatively less hardness during the baking of the cookies was observed [27,34]. An evident correlation (R^2^ = 0.61) was observed between the specific volume and hardness of the cookies. From the results in Table 1, it is clear that the higher the specific volume was for a cookie, the lower the hardness.

A second quality criterion of the texture profile analysis for a cookie is determined by its springiness. A product’s physical ability to physically spring back after being deformed during the initial compression is known as springiness [35]. The conventional cookie, as shown in Table 1, had the highest springiness, 0.78 ± 0.03%, whereas the standard cookie produced under the new conditions, 0.76 ± 0.09%, had the second-highest springiness. The cookies baked without a leavening agent displayed the least amount of springiness for both baking conditions. All the GH-containing cookie samples, meanwhile, had a similar and lower-than-average percentage of springiness.

The percentage of pores in the area in mm^2^ was the next quality criterion to be examined (Table 2). The bubbles first grow by the chemical or biological production of carbon dioxide and then later through evaporation and gas expansion brought on by the baking heat. Both the inclusion and stability of gas bubbles are essential for achieving a low crumb density [35]. Furthermore, a 20% increase in porosity results in a 7 min reduction in the baking time because the heat transmission during baking greatly depends on the product’s gas volume percentage [36,37]. Hence, the improvised kneading with less baking time was seen to improve the porosity of the cookie by comparing it with cookies made under normal conditions. The pore analysis software categorized the quantity of the pores into five groups based on their size, which ranged from extremely small to extremely large. For cookies, it is ideal to have a uniform distribution of extremely small and small pores at a maximum percentage. The number of large pores is recommended to be little as possible. Table 2 demonstrates that GH-made cookies had larger pores with respect to ammonium bicarbonate-made cookies. When GH was employed as a leavening agent in the cookie dough, the unequal dispersion of gas molecules could be understood as the cause of this.

### 3.2. Colour and Appearance of Black-and-White Cookies

**Figure 2:**
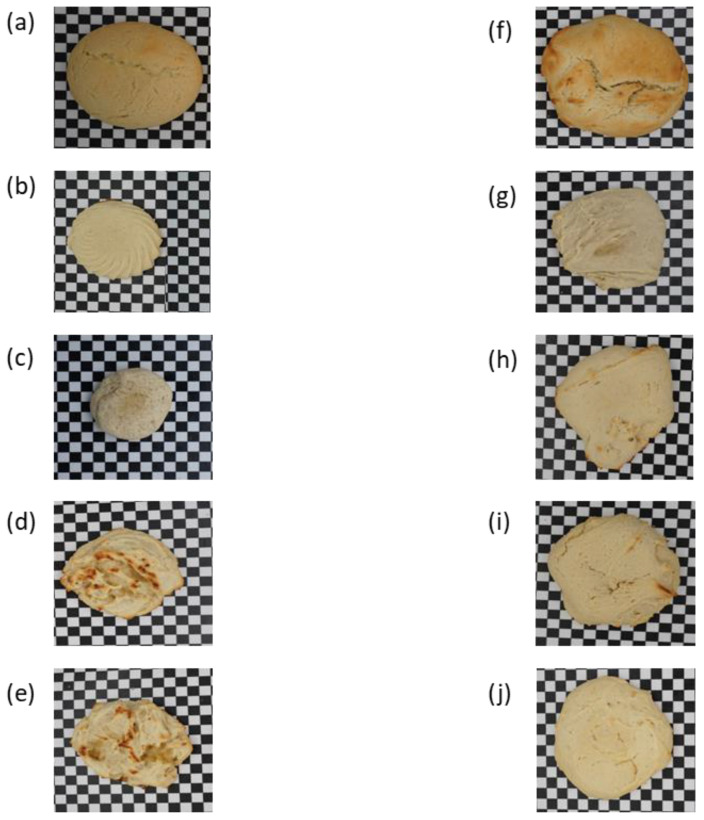
Pictures of black-and-white cookies made under different conditions. (**a**) Standard cookies, (**b**) Cookies with no leavening agent, (**c**) Cookies made with 20% GH, (**d**) Cookies made with 40% GH, (**e**) Cookies with 50% GH, (**f**) Standard cookies made with high temperature kneading (HTK), (**g**) Cookies with no leavening agent made with HTK, (**h**) Cookies with 20% GH made with HTK, (**i**) Cookies with 40% GH made with HTK, (**j**) Cookies with 50% GH made with HTK.

Figure 2 reveals a noticeable change in the color as well. From Table 3, the standard cookies had a minimal L* value which represented their lightness of color when compared to the ones without ammonium bicarbonate. As the L*a*b* space and L* value typically decreased, where a* and b* values increased for biscuits baked in the conventional process, this significantly increased the amounts of acrylamide formed in the biscuits during conventional baking [38]. The higher baking temperature caused the intense browning of the cookies produced under an improvised technique [39]. However, in both baking scenarios, ammonium bicarbonate-based cookies always had an intense brown color with no significant difference from each other. This could be explained by the addition of ammonium bicarbonate, which lowers pH, and how Maillard’s reaction is affected by a low pH. The Maillard reaction and caramelization are the main chemical reactions that cause the browning of cereal-based products during baking. Both procedures are reliant on the physicochemical properties of the dough, such as the levels of reducing sugar, amino acids, water activity, and pH, as well as the baking circumstances, such as temperature, airflow, and relative humidity [40]. As a result, it is always noticeable that cookies made with GH and those without a leavening agent are lighter in color than typical cookies. Browning is significantly influenced by the ammonium bicarbonate concentration as the Maillard reaction is accelerated by ammonia which causes an increase in pH [41].

### 3.3. Acrylamide Content in Black-and-White Cookies

**Figure 3:**
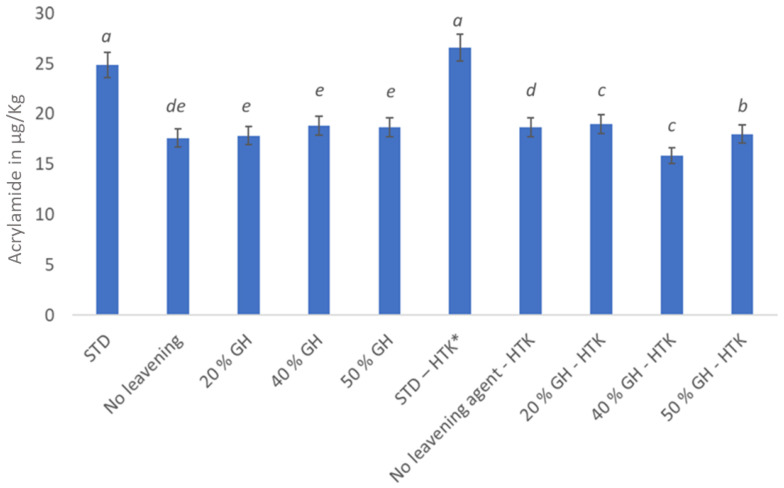
Acrylamide content in µg/Kg of the sample of black-and-white cookies baked under different conditions. Values with the same superscripts did not differ significantly (*p* ˂ 0.05). * HTK—high temperature kneading.

If ammonium bicarbonate and sugar were present, acrylamide production at 60 °C was found in numerous systems [42]. From Figure 3, it can be seen that acrylamide was present in all cookies in different quantities. However, no significant difference could be observed between the standard cookies baked under normal and improvised conditions. According to Amrein [41], within the first 15 min, the acrylamide concentrations at 200 °C were hardly higher than those at 180 °C. The amount of acrylamide increased even more when the baking procedure continued past the 10 min point. Therefore, to reduce the amount of acrylamide, lengthy baking or extreme browning should be avoided. In general, it was found that extended baking at lower temperatures led to a content of acrylamide that was much higher, as seen in Figure 3 [43]. This could also be one of the reasons, other than the presence of ammonium bicarbonate, for the increased acrylamide content in both the standard cookies. In order to prevent the production of acrylamide, baking at higher temperatures for a shorter period of time is preferable. Additionally, the baking time could be decreased by raising the baking temperature when GH is used instead of ammonium bicarbonate. This showed a significant decrease in the quantity of acrylamide, as shown in Figure 3. This discovery lends support to the novel baking procedures used to create black-and-white cookies.

## 4. Conclusions

One of the challenges that this research attempted to solve was the use of CO_2_ GH as a leavening agent for black-and-white cookies in the baking business. High-temperature kneading was required for GH gas solubility to combine appropriately in a dough with high-fat content. This was because the GH changed the texture of the cookie dough, which caused a sudden reduction in temperature which was one of the greatest challenges that this study faced. The best results of this study were obtained while using 50% GH and kneading the dough at a high temperature, as opposed to baking regular cookies, while the results achieved with a lower GH percentage and utilizing standard kneading techniques revealed poor quality cookies. When manufactured with GH by HTK, the specific volume was likewise within an acceptable range. This demonstrated almost proper pore development, which allowed moisture to escape during the baking process. The range of results for the moisture content of the cookies is similar to the normal range. Cookies containing GH had larger pores overall due to the unequal distribution of gas molecules. Cookies manufactured with GH were similar to those made with ammonium bicarbonate in terms of their hardness and springiness. Additionally, and most importantly, the acrylamide content was reduced considerably by substituting ammonium bicarbonate with GH. However, more research is needed to produce cookies with the same quality as regular black-and-white cookies.

This study has clarified the challenges in relation to GH implementation in the baking sector and has laid the groundwork for the creation of fresh strategies to overcome these challenges. Due to the higher energy consumption, high-temperature kneading may face difficulties. The main benefit, however, is that GH is clearly labeled, and the final product contains fewer dangerous ingredients, such as acrylamide. With the elimination of ammonium bicarbonate, a 27% reduction in the total acrylamide of black-and-white cookies was obtained. The next phase of this experiment could involve applying GH to additional baked items. The incorporation of GH into the dough might be studied using a kneader that operates in the headspace, under a vacuum, or with a headspace gas regulating system in the future. It is feasible to create gas hydrates on-site by putting a reactor in the bakery. The GH reactor might be modified to produce uniform, small particle-sized GH, which could help overcome challenges and shorten the time needed for high-temperature kneading. A study on the partial replacement of ammonium bicarbonate by GH and its process optimization could have the potential for a forthcoming study in related research.

## Figures and Tables

**Table 1:** Physical characteristics of the cookies baked under different conditions.

	Specific Volume (mL/g)	Moisture (%)	Hardness (N)	Springiness (%)
STD	2.85 ± 0.04 *^a^*	30.6 ± 0.3 *^a^*	5 ± 1.08 *^g^*	0.73 ± 0.13 *^a^*
No leavening	0.74 ± 0.02 *^de^*	30.12 ± 0.31 *^a^*	90 ± 4.12 *^a^*	0.52 ± 0.01 *^c^*
20% GH	0.719 ± 0.02 *^e^*	30.67 ± 0.23 *^a^*	45.52 ± 4.26 *^cd^*	0.71 ± 0.03 *^a^*
40% GH	0.724 ± 0.03 *^e^*	30.75 ± 0.19 *^a^*	39.08 ± 1.65 *^d^*	0.74 ± 0.03 *^a^*
50% GH	0.73 ± 0.01 *^e^*	31.10 ± 0.62 *^a^*	38. 76 ± 1.89 *^d^*	0.73 ± 0.01 *^a^*
STD—HTK *	2.75 ± 0.06 *^a^*	29.58 ± 0.42 *^b^*	8.41 ± 0.88 *^f^*	0.76 ± 0.09 *^a^*
No leavening agent—HTK	0.89 ± 0.11 *^d^*	30.12 ± 0.18 *^a^*	70.36 ± 2.14 *^b^*	0.56 ± 0.08 *^b^*
20% GH—HTK	1.40 ± 0.09 *^c^*	31.57 ± 0.76 *^a^*	48.94 ± 4.43 *^c^*	0.70 ± 0.07 *^a^*
40% GH—HTK	1.44 ± 0.06 *^c^*	30.82 ± 0.58 *^a^*	40.81 ± 3.41 *^cd^*	0.72 ± 0.05 *^a^*
50% GH—HTK	1.80 ± 0.05 *^b^*	30.76 ± 0.41 *^a^*	31.25 ± 2.18 *^e^*	0.73 ± 0.02 *^a^*

Values are expressed as the means of triplicate samples ± standard deviation (*n* = 3). Values with the same superscripts in a column did not differ significantly (*p* ˂ 0:05). * HTK—high temperature kneading.

**Table 2:** The percentage of pore sizes mm^2^ in cookies baked at different conditions.

	Very Small	Small	Medium	Big	Very Big
STD	36.44 ± 4.87 *^de^*	11.7 ± 1.87 *^ab^*	27.29 ± 2.45 *^a^*	20.93 ± 3.56	3.67 ± 1.41 *^ab^*
No leavening	39.01 ± 3.98 *^bcd^*	12.71 ± 2.07 *^ab^*	24.93 ± 2.59 *^ab^*	19.26 ± 2.76	4.1 ± 1.56 *^ab^*
20% GH	43.62 ± 3.92 *^a^*	10.75 ± 2.05 *^ab^*	22.40 ± 2.64 *^ab^*	19.99 ± 3.82	3.24 ± 1.74 *^b^*
40% GH	38.74 ± 4.68 *^cd^*	14.83 ± 3.31 *^a^*	19.22 ± 2.87 *^b^*	18.65 ± 2.65	8.56 ± 1.63 *^a^*
50% GH	43.97 ± 5.52 *^a^*	9.9 ± 1.54 *^b^*	20.2 ± 2.66 *^b^*	24.31 ± 3.69	1.61 ± 0.98 *^b^*
STD—HTK *	41.79 ± 2.02 *^abc^*	10.43 ± 1.25 *^ab^*	22.31 ± 2.13 *^ab^*	22.07 ± 3.73	3.39 ± 1.22 *^ab^*
No leavening agent—HTK	42.80 ± 3.72 *^a^*	9.71 ± 2.16 *^b^*	23.03 ± 1.92 *^ab^*	20.42 ± 2.88	4.06 ± 1.08 *^ab^*
20% GH—HTK	31.35 ± 3.94 *^f^*	13.34 ± 2.77 *^ab^*	24.91 ± 1.66 *^ab^*	24.66 ± 3.49	5.73 ± 2.26 *^a^*
40% GH—HTK	32.65 ± 2.13 *^f^*	12.59 ± 1.65 *^ab^*	26.07 ± 0.59 *^a^*	21.64 ± 3.01	7.05 ± 2.99 *^a^*
50% GH—HTK	35.71 ± 4.36 *^e^*	10.6 ± 2.75 *^ab^*	25.3 ± 1.94 *^ab^*	22.37 ± 2.74	6.02 ± 1.15 *^a^*

Values are expressed as the means of triplicate samples ± standard deviation (*n* = 3). Values with the same superscripts in a column did not differ significantly (*p* ˂ 0.05). No significant difference observed in the data of big pores. * HTK—high temperature kneading.

**Table 3:** L*a*b values of black-and-white cookies baked at different conditions.

	L*	a*	b*
STD	76.78 ± 1.17 *^c^*	1.70 ± 0.82 *^ab^*	13.2 ± 2.12 *^b^*
No leavening	84.08 ± 0.67 *^a^*	0.8 ± 0.01 *^b^*	12.04 ± 0.81 *^b^*
20% GH	81.86 ± 0.48 *^ab^*	1.6 ± 0.93 *^ab^*	12.21 ± 3.52 *^b^*
40% GH	83.97 ± 1.06 *^ab^*	2.15 ± 0.31 *^a^*	12.95 ± 0.99 *^b^*
50% GH	82.16 ± 1.65 *^ab^*	2.37 ± 0.43 *^a^*	14.26 ± 1.34 *^b^*
STD—HTK *	76.28 ± 1.00 *^c^*	3.54 ± 1.05 *^a^*	20.47 ± 1.03 *^a^*
No leavening agent—HTK	79.90 ± 1.53 *^b^*	0.71 ± 0.04 *^b^*	12.59 ± 0.59 *^b^*
20% GH—HTK	82.44 ± 0.92 *^ab^*	0.89 ± 0.13 *^b^*	11.81 ± 0.74 *^b^*
40% GH—HTK	77.15 ± 0.76 *^c^*	1.24 ± 0.04 *^b^*	15.32 ± 0.39 *^b^*
50% GH—HTK	81.58 ± 1.95 *^ab^*	0.83 ± 0.65 *^b^*	15.08 ± 0.61 *^b^*

Values are expressed as the means of triplicate samples ± standard deviation (*n* = 3). Values with the same superscripts in a column did not differ significantly (*p* ˂ 0.05). * HTK—high temperature kneading.

## Data Availability

The data used to support the findings of this study can be made available by the corresponding author upon request. (In case of special circumstances please refer to the following links: https://www.mdpi.com/journal/foods/instructions accessed on 20 June 2023).
